# UV-Induced Nanoparticles-Formation, Properties and Their Potential Role in Origin of Life

**DOI:** 10.3390/nano10081529

**Published:** 2020-08-04

**Authors:** Lukas Nejdl, Kristyna Zemankova, Martina Havlikova, Michaela Buresova, David Hynek, Kledi Xhaxhiu, Filip Mravec, Martina Matouskova, Vojtech Adam, Martin Ferus, Jakub Kapus, Marketa Vaculovicova

**Affiliations:** 1 Department of Chemistry and Biochemistry, Mendel University in Brno, Zemedelska 1, CZ-613 00 Brno, Czech Republic; lukasnejdl@gmail.com (L.N.); zemankova.kristyna@seznam.cz (K.Z.); xbureso6@mendelu.cz (M.B.); d.hynek@email.cz (D.H.); martinka.matouskova@gmail.com (M.M.); vojtech.adam@mendelu.cz (V.A.); 2 Central European Institute of Technology, Brno University of Technology, Purkynova 123, CZ-612 00 Brno, Czech Republic; 3 Materials Research Centre, Faculty of Chemistry, Brno University of Technology, Purkynova 118, CZ-612 00 Brno, Czech Republic; xchavlikovam@fch.vut.cz (M.H.); mravec@fch.vut.cz (F.M.); 4 Department of Chemistry, Faculty of Natural Sciences, University of Tirana, Blv. Zog I, Nr. 2/1, 1001 Tirana, Albania; kledi.xhaxhiu@fshn.edu.al; 5 J. Heyrovsky Institute of Physical Chemistry, Czech Academy of Sciences Dolejskova 3, CZ-182 23 Prague 8, Czech Republic; martinferus@email.cz; 6 Slovak Organisation for Space Activities, Zamocka 5, 811 03 Bratislava, Slovakia; jakub.kapus@sosa.sk

**Keywords:** UV-induced nanoparticles, origin of life, metal ions, thiols, nanoparticle-based world, nanozymes

## Abstract

Inorganic nanoparticles might have played a vital role in the transition from inorganic chemistry to self-sustaining living systems. Such transition may have been triggered or controlled by processes requiring not only versatile catalysts but also suitable reaction surfaces. Here, experimental results showing that multicolor quantum dots might have been able to participate as catalysts in several specific and nonspecific reactions, relevant to the prebiotic chemistry are demonstrated. A very fast and easy UV-induced formation of ZnCd quantum dots (QDs) with a quantum yield of up to 47% was shown to occur 5 min after UV exposure of the solution containing Zn(II) and Cd(II) in the presence of a thiol capping agent. In addition to QDs formation, xanthine activity was observed in the solution. The role of solar radiation to induce ZnCd QDs formation was replicated during a stratospheric balloon flight.

## 1. Introduction

Nanozymes are modern, man-made alternatives to natural enzymes. Their main advantages over the natural enzymes include stability, scalability, chemical diversity, and functionality in nonaqueous solvents [1]. Nowadays, nanozymes, the next generation of artificial enzymes, are made especially from metal or carbon materials [2]. Indeed, quantum dots (QDs) appear to be suitable for this purpose because of their properties by changing their size, shape, spatial arrangement and surface modification. The versatility of QDs consist not only in a wide range of application but also in many approaches of aqueous syntheses assisted by microwaves [3], lasers [4], UV irradiation [5], electrochemistry [6,7], ultrasounds [8] or living organisms [9] (microorganisms, animals, plant extract, fungi and actinomycete) and also all precursors of QDs like Zinc, Cadmium and Sulfur can be found in the same ore—Sphalerite or Wurtzite [10]. This enormous versatility nominates them as suitable models relevant to the prebiotic chemistry. QDs display not only size-dependent characteristics (quantum confinement), but it was also proved that CdTe QDs specifically recognize and cleave (both in vitro and in vivo) the restriction site GAT′ATC (′ = cleaving site) in double-stranded DNA upon light irradiation [11]. The similar RNA analogue (where T = U) of these DNA restriction sites (GATATC) can be found in RNA [12]. These are examples of reactions involving nanoparticles. The formation of nanoparticles can be initiated under a variety of conditions and it is likely that these conditions may have existed on planet Earth at a certain point after its formation based on generally accepted assumptions, as some of such conditions may have been present on planet Earth at a certain point after its formation. Certain nanoparticles formed under these conditions might have served as initiators or surfaces enabling reactions leading to a transition from inorganic chemistry to self-sustaining living networks. Numerous reactions might have been mediated, controlled and driven by nanoparticles and therefore, the synthesis of simple organic molecules with potential biological activity has started.

Nanoparticles in the role of enzymes (nanozymes) open up a completely new view on the origin of life theory. The enzymatic activity of inorganic materials has begun to be recognized in the context of new theories of the origin of life [13,14]. Many theories, such as the Metabolism-first world [15], Zinc world [16], Thioester world [17], RNA world [18] and others [19], are currently being proposed as possible explanations for the origins of life.

The Metabolism-first world scenario is built on a premise that any living entity can metabolize nutrients, harvest energy, and use it to sustain itself. In one version, it proposes that life originated from a simple metabolism-like prebiotic arrangement, in which iron sulfide minerals catalyzed reactions in a reverse Krebs-like cycle [20]. Evans et al. have provided evidence of this ferredoxin-dependent carbon-reduction cycle in a photosynthetic bacterium [21]. Wächtershäuser, who attempted to reproduce this cycle by coupling oxidation of FeS into FeS_2_ (pyrites) with CO_2_ fixation and reduction, has provided a simplified version of this system [22]. The cycle operates to a certain extent and produces at least CH_3_SH as a form of partially reduced carbon and reactive species. For this reason, the Iron-sulfur world has received mixed reactions as a possible Metabolism-first theory.

The Zinc world scenario presumes that carbon dioxide was reduced in a solar-driven reaction on ZnS surfaces, yielding building blocks of the first biopolymers. Subsequently, these compounds served as templates for the synthesis of larger biopolymers. The attraction of the Zinc-sulfur would is obvious, but no attempt as yet has been made to mimic biological processes. This scenario is envisaged to operate on mineral surfaces exposed to a stellar (in the case of early Earth) radiation, which drives the reaction. However, the ZnS surfaces also protect the first reaction products from photodissociation by absorbing the excess of incoming radiation [16].

Christian de Duve proposed the Thioester world where coupling a thiol with carboxyl-containing molecules under acidic and high-temperature conditions produces thioesters that can activate amino acids and polymerize to form oligomers. Some of these oligomers exhibit catalytic activity similar to that of major enzyme classes. A primitive metabolic network can then be envisaged, where the activated thioesters provide a source of energy and the amino acid oligomers supply the catalytic function. Such a scenario may have been common in, for example, hydrothermal vents [17].

According to RNA world theory, the first living systems were “naked” self-replicating RNA molecules, without any membrane, mediating biochemical reactions and transferring the genetic information [23]. The functions of ribozymes in modern organisms support the hypothesis that life passed through an RNA world before the emergence of proteins or DNA. The functions of ribozymes in modern organisms support the hypothesis that life passed through an RNA world before the emergence of proteins or DNA. Indeed, many present day microorganisms can produce nanoparticles as a result of metal detoxification pathways associated with sulfur metabolism [24,25]. This ability of microorganisms can also support the hypothesis that life passed through the nanoparticles-assisted stage.

In this work, a new possibility for a prebiotic scenario based on UV-induced formation of metal nanoparticles is proposed. It has been demonstrated that such nanoparticles could have been conceivably produced on early Earth and synthesized from a simple mixture of thiols (mercaptosuccinic acid, MSA), Zn(II) and Cd(II) ions exposed to UV radiation.

## 2. Materials and Methods

### 2.1. Chemicals 

Standards and other chemicals were purchased from Sigma-Aldrich (St. Louis, MO, USA) in ACS purity.

### 2.2. Thiols, Metals, Ions Standards and Phosphate Buffer Preparation

Stock solutions of 16 mM MSA, 10 mM zinc(II) acetate (Zn(II)), 10 mM cadmium(II) acetate (Cd(II)), 30% ammonia (NH_3_) and 100 mM phosphate buffer pH 7 were prepared in distilled water and stored at 4 °C.

### 2.3. Solution Preparation

Solutions of 6 mM zinc(II) acetate, 6 mM cadmium(II) acetate, 16 mM MSA, and 100 mM phosphate buffer pH 7 were prepared. 43 μL of zinc(II) acetate, 7 μL of cadmium(II) acetate, 25 μL of MSA and 25 μL of phosphate buffer pH 7 were pipetted into the UV-transparent 96 well plate with a flat bottom by CoStar (Corning, KS, USA). Subsequently, the plate was placed into the UV transilluminator (Vilber Lourmat, Marne-la-Vallee Cedex, France) with λ_em_ = 254 nm and irradiated for 0, 1, 2, 4, 8, 16, and 32 min. The sample area of 20 × 20 cm was illuminated by 6 emitting tubes with a power of 15 W each. Subsequently, the wavelength of 250 nm was used for excitation and the fluorescence scan was measured within the range from 300 to 800 nm by Tecan Infinite 200 M PRO (TECAN, Switzerland).

### 2.4. Layout of the Experiment

An ammonia solution was prepared by dilution of NH_3_ in distillate water in range of 15%, 7.5%, 3.8%, 1.9%, 1%, 0.5%, 0.2 and 0%. The solution of 6 mM Zn(II) in volume range 48 to 2 μL and solution of 6 mM Cd(II) in volume range 2 to 48 μL were mixed in a 96 well plate and 25 μL of 16 mM MSA was added into each well. Additionally, 25 μL of 15% to 0% NH_3_ was added and therefore, the total volume of precursors (Zn:Cd:MSA:NH_3_) in each well was 100 μL. A detailed composition of each solution is shown in Tables S1 and S2. Afterwards, the plates with precursors were irradiated and monitored by a camera in time range 0–14 min. The video of the QD formation process can be found in Supplementary Information.

### 2.5. Characterization of Size and Zeta Potential of ZnCd QDs

The zeta potential and size of particles were measured by dynamic light scattering using the Zetasizer Nano ZS instrument (Malvern Instrument Ltd., UK). The parameters of particle size measurements were: refractive index of the dispersive phase of 3.00 and 1.333 for the dispersive environment, adsorption coefficient 10^−3^, temperature 25 °C, equilibration time 120 s, measurement angle of 173° backscatter. For measurement, disposable cuvettes, type ZEN 0040 containing 50 µL of sample were used. The zeta potential measurement parameters such as temperature and equilibrating time were the same as in particle size measurements. Calculations considered the diminishing of particles concentration-based Smoluchowski model, with F(κa) of 1.50. For measurement, disposable cuvettes, type DTS1070, were used. The measurements were performed under the automatic setting of attenuation and voltage selection. All measurements were carried out in triplicates.

### 2.6. Transmission Electron Microscopy (TEM)

The precursors (Zn:Cd:MSA in 100 mM phosphate buffer pH 7) were irradiated for 8 min and formed ZnCd QDs were subsequently precipitated by iso-propanol and resuspended in distilled water. TEM micrographs of dried samples on copper grids were taken with a Tecnai F20 microscope (FEI, Netherlands) at appropriate magnifications.

### 2.7. Determination of Quantum Yield of ZnCd QDs 

The quantum yield (QY) was calculated for QDs prepared from precursors Zn:Cd:MSA in 100 mM phosphate buffer pH 7 (4 min irradiation by UV transilluminator). The QYs were determined by analysis of emission maxima with the excitation at λ = 360 nm. The absorbance was analyzed using the same wavelength (λ = 360 nm). The quantum yields were calculated based on integration of absorption and emission value. Absolute values were calculated using the standard samples (quinine sulphate), with known fluorescence QY value, according to Equation (1):

(1) $$Ø\;X=Ø\;ST\left(\frac{Gradx}{GradST}\right)\left(\frac{\eta^{2}x}{\eta^{2}ST}\right)$$

where the subscripts *ST* and *X* denote standard and test, respectively, *Ø* is the fluorescence quantum yield (*Ø ST* = 0.54), *Grad* is the gradient from the plot of integrated fluorescence intensity vs. absorbance (*Grad ST* = 80,000,000, and *η* the refractive index of the solvent (1.333)).

### 2.8. Enzymatic Reactions

(1) To monitor the generation of superoxide anion from MSA (i.e., photooxidation) during UV irradiation. Superoxide dismutase assay kit from Sigma-Aldrich (St. Louis, MO, USA) was used. Highly water-soluble tetrazolium salt that produces a formazan dye upon reduction with a superoxide anion was used. This reaction was monitored at 440 nm by UV/Vis spectrophotometer and was proportional to the amount of superoxide anion.

(2) Xanthine oxidase-like activity of ZnCd QDs after visible light irradiation was investigated also by highly water-soluble tetrazolium salt that produces a formazan dye upon reduction when a superoxide anion was used. This reaction was monitored at 440 nm by UV/Vis spectrophotometer and was proportional to the amount of superoxide anion.

## 3. Results and Discussion

Although oxygen is the third most abundant element in the Universe by mass [26], and on the modern Earth, it is the most widespread element, the primordial atmosphere very likely lacked molecular oxygen, being present in five orders of magnitude lower concentration than today [27]. Thus, it is hypothesized that the primordial environment had a reducing character [28]. The degree to which the atmosphere was reducing is still uncertain and the exact composition of the reducing mixture remains unknown.

Extraterrestrial impacts of asteroids and meteorites, rich in heavy metals and ammonia, could deliver water, atmospheric gases and organic refractory material from outer colder regions of evolving protoplanetary disks to the early Earth’s surface. They also certainly affected the mineral composition of early Earth’s surface and its anoxic chemical environment, in which prebiotic synthesis was conceived. Equally, sulfur chemistry was typical for hydrothermal vents on early Earth. In some impact structures, the metal content was enhanced with the addition of thiols and NH_3_ [29]. These structures were also subjected to alteration by liquid water. UV radiation of a young Sun emitting stronger UV/XUV/X-ray fluxes than older evolved stars was even enhanced [30]. Additionally, the total amount of UV-B and UV-C radiation that reached the surface of early Earth in the Archean age might have been higher than nowadays, and further enhanced due to the lack of an ozone layer [31] which might have led to the formation of various types of metallic nanoparticles or nanoclusters ([2Fe–2S] and [4Fe–4S] [32]) with nanozymatic or redox activity. Principles involved in the behavior of these nanoclusters/nanoparticles might have evolved into metabolic-like networks preceding the emergence of the first living systems. In our study, the formation of ZnCd QDs with interesting photophysical behavior in phosphate or NH_3_ environment was explored not only under laboratory conditions but also during stratospheric balloon flight simulating harsh radiation on the surface of early planets exposed to strong UV flux of early parent stars and lacking, at the same time, ozone layer in their primary atmospheres. 

### 3.1. Formation of ZnCd QDs

It was proved that simple redox reactions associated with metal ions (Zn(II) and Cd(II)) and thiols (e.g., MSA) driven by UV radiation result in the one-pot synthesis of highly fluorescent (QY = 47%) ZnCd QDs. The environment solution was controlled by phosphate buffer because there is a direct implication about life. In general, phosphate anions play an important role in maintaining acid-base balance in organisms and phosphate also formed a sugar-phosphate backbone in DNA/RNA.

**Figure 1:**
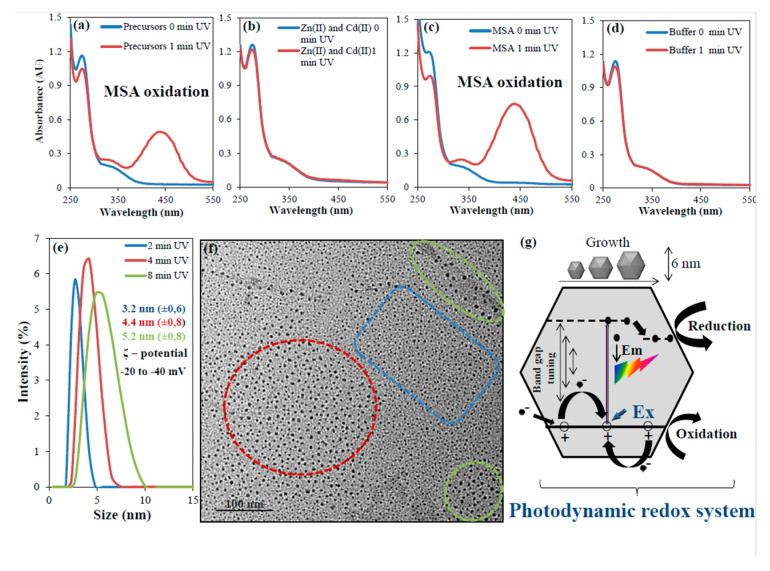
Monitoring of behavior of individual components of the precursor mixture. UV/Vis absorption spectra of tetrazolium salt producing a formazan dye upon reduction by a superoxide anion generated by photodissociation (photo-oxidation) of thiols (MSA) before (blue) and after (red) 1 min UV irradiation, the sample consisted from precursors (**a**) Zn(II), Cd(II) and MSA in pH 7 controlled by phosphate buffer, (**b**) Zn(II), Cd(II) in phosphate buffer pH 7, (**c**) solution of MSA in phosphate buffer pH 7 and (**d**) phosphate buffer pH 7 solution. QDs (quantum dots) characterization according to (**e**) size distribution of ZnCd QDs in range 3–5 nm after 2 (blue), 4 (red) and 8 (green) min UV irradiation and respective zeta potential in range the −20 to −40 mV, (**f**) Transmission electron micrograph of ZnCd QDs after 8 min UV irradiation of precursors (Zn:Cd:MSA in phosphate buffer pH 7), scale bar represents 100 nm. (**g**) Schematic representation of the proposed photodynamic redox system. Excited electrons (-) have a strong reduction potential and holes (+) are very strong oxidizing agents. Oxidation-reducing properties of ZnCd QDs can be significantly changed according to the band gap distance (↔) associated with growing of QDs.

The formation of ZnCd QDs was studied by evaluation of redox properties of the mixture of precursors and corresponding individual components. The behavior was monitored at λ = 440 nm and was proportional to the amount of superoxide anion (RSH + O_2_^UV^→ RS+ O^•^_2_). The mixture of precursors containing Zn(II), Cd(II) and MSA in phosphate buffer pH 7 (Figure 1a), a mixture of metal ions containing Zn(II), Cd(II) in phosphate buffer pH 7 (Figure 1b), solution of MSA in phosphate buffer pH 7 (Figure 1c), and phosphate buffer pH 7 solution (Figure 1d) were analyzed by absorption spectrometry before (0 min) and after 1 min UV irradiation. A strong photooxidation of MSA was observed as a gradual increase of the absorption peak located around 440 nm (Figure 1a–c). In case of a mixture of metal ions containing Zn(II), Cd(II) in phosphate buffer pH 7 (Figure 1b) and phosphate buffer pH 7 solution (Figure 1d), no significant change was observed. It was proved that the photooxidation of MSA is necessary for Zn(II) and Cd(II) coordination, leading to the formation of ZnCd QDs. Oxygen-poor and probably reducing environment of prebiotic Earth [33] certainly harbored lower oxidation states of prebiotically important compounds of phosphorus and sulfur delivered by meteorite [34]. Alternatively, others such as phosphonic (H_3_PO_3_), phosphinic (H_3_PO_2_), [35] and sulfonic acids (R−SO_2_OH) and their certainly more stable derivatives might have been produced by volcanic activity [36].

Next, mixture of precursors (Zn:Cd:MSA with phosphate buffer pH 7) was irradiated for different times (2–8 min). Using a dynamic light scattering analysis, it was proved that the studied mixture contained 3.2, 4.4, and 5.2 nm ZnCd QDs particles depending on irradiation time (2, 4, and 8 min) with zeta potential in the range −20 to −40 mV, Figure 1e. The zeta potential of ZnCd QDs suggested that the suspension was stable, because particles with zeta potential above/below ± 30 mV are generally considered to be stable in suspension [37]. Micrographs obtained by transmission electron microscopy also confirmed the polydispersity of ZnCd QDs suspension, Figure 1f. This finding is in agreement with the generally accepted theory of size-dependent properties of QDs [38]. A fluorescence mapping (fluorescence intensity as a function of the excitation wavelength after 5 min UV irradiation of precursors) of ZnCd QDs is shown in Figure S1. It was also shown that band gap tuning can depend on irradiation time or MSA concentration. Longer irradiation time generates larger nanoparticles with smaller band gaps (Figure S2a). The nanoparticle band gap and diameter can also be modulated by the MSA concentration. The fluorescence maximum shifted to higher wavelengths when the MSA concentration increased, which indicates a smaller band gap, Figure S2b. The relationship between particle size and band gap size is schematically shown in Figure S2c. Finally, we tested the influence of pH in range 6–9 controlled by phosphate buffer. Fluorescence was stable in most cases, only pH 6 caused a decrease of the fluorescence intensity by approximately 28% (data not shown).

Under conditions described above, ZnCd QDs are formed in one pot after 2–8 min of irradiation. UV radiation induces a strong thiol group photodissociation (oxidation). This reaction initializes the formation of ZnCd QDs because thiols (R-SH) are changed to thiolates (R-S^−^), thiyls (R-S·) or disulphides (R-S-S-R). Thiolates are strong nucleophiles because they can donate three electron pairs to an electrophile (Zn(II) and Cd(II)) and form a donor-acceptor chemical bond and can be stabilized by disulphides. Disulphides and thiols can provide two electron pairs to Zn(II)/Cd(II) coordination. It is important to note that:

(a) ZnCd QDs are formed by redox reactions during photodissociation/photooxidation of thiol-containing compounds with Zn(II) and Cd(II) in a short time,
(b) ZnCd QDs exhibit strong fluorescence (QY = 47%),
(c) ZnCd QDs grow upon UV irradiation (band gap tuning).

Heterogeneous catalysis is one of the most important chemical processes of various industries performed on catalyst nanoparticles with different sizes or/and shapes [39,40]. Similarly, a heterogeneous photocatalysis is a process taking place on various surfaces in case of absorption of radiation with the same or higher energy as is the energy of the band gap. The redox reactions and emission properties (fluorescence) of ZnCd QDs are strictly dependent on the size of the band gap (eV). The band gap tuning (ZnCd QDs grow up) is associated with the time of UV irradiation. Based on these results, it was proposed to call this set of redox reactions a photodynamic redox system (Figure 1g).

These fascinating reactions could have taken place under prebiotic conditions and could have been driven by solar radiation. To verify the ability of natural solar radiation to induce the formation of ZnCd QDs, a stratospheric probe (balloon) was launched with a set of spectroscopic cuvettes filled by ZnCd QDs precursors (Zn:Cd:MSA). The scheme of the setup is shown in Figure S3a. Three cuvettes were exposed UV irradiation and two cuvettes were shielded from UV irradiation inside a probe. The flight trajectory is shown in Figure S3b to demonstrate that the overall flight duration was 2 h and 17 min and the maximum altitude reached was 29,434 m.

It is clearly shown by emission spectra that solar radiation induced ZnCd QDs from precursors (Figure S3c).

### 3.2. Influence of Ammonia (NH_3_) Environment on ZnCd QDs Emission Properties 

Pizzarello et al. found large amounts of NH_3_ in an Antarctic asteroid and they assumed that high concentration of NH_3_ could account for a sustained source of reduced nitrogen essential to the origin of life chemistry [41]. Other sources of reduced nitrogen proposed so far include photochemical reduction of N_2_ to NH_3_ in the presence of titanium oxide, hydrogen cyanide or formamide production in a CO_2_/N_2_ atmosphere via the action of UV radiation and direct reduction of N_2_ to NH_4_^+^ in aqueous solution catalyzed by Ni/Fe metals and iron sulfides [42,43]. An abundant exogenous and endogenous of NH_3_, therefore, might have been significant in aiding early Earth molecular evolution which is in line with our Nanoparticle world theory.

**Figure 2:**
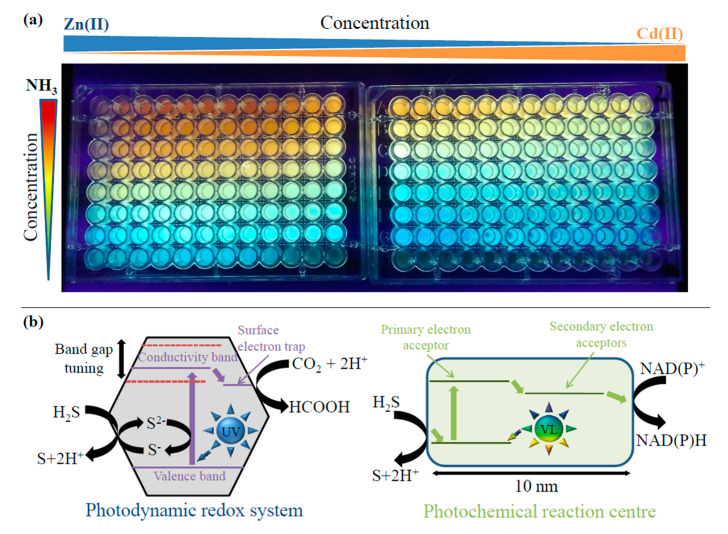
(**a**) Photograph of ZnCd QDs after 8 min UV irradiation of precursors (Zn:Cd:MSA:NH_3_). (**b**) Schematic representation of photodynamic redox system in comparison with bacterial photochemical reaction center adapted from [46].

It was proved that different NH_3_ concentrations together with different ratios of metal ions (Zn(II) and Cd(II)) significantly influence the emission properties of ZnCd QDs during UV irradiation, as can be seen in Figure 2a. Emissions of QDs (rainbow colors) are due to the varying ratio of precursors. The concentrations, as well as volumes of NH_3_ and the metal ion ratios are listed in Table S1 and S2. The video showing UV-induction of ZnCd QDs in real-time (0–14 min irradiation) can be also seen in the Supplementary Information. This approach can be used to prepare ZnCd QDs covering the whole spectral range (emission from blue to red) which can be prepared. The electronic (resonance) transition between QDs valence and conductivity band energy levels resembles the electron transfer observed in various natural/biological systems, such as in light-harvesting complexes and photochemical reaction centers of living cells [44]. For example, Hongjin et al. used multicolor CdSe QDs for enhanced light-harvesting ability under white light of light-emitting diode [45].

The proposed theory that nanoparticles assisted in the origin of some key (bio)molecules presumes that the feasibility of reactions occurring at nanoparticle surfaces depends on the band gap, conductivity band position, and surface electron trap location, Figure 2b (left). The oxidation-reduction properties of the nanoparticles can dramatically change according to the band gap distance. Absorption of UV quantum leads to the separation of electric charges. The electrons migrate in the ZnCd QDs until they are trapped at the surface. The trapped electrons can reduce the CO_2_ molecule either via two-electron transfer or possibly in a concerted two-electron reaction. The electron vacancy (hole) is initially reduced by the S^2-^ ion of the ZnCd QDs. The ultimate electron equilibration requires external electron donors from H_2_S [16]. Figure S4a compares standard redox potentials and band gap energies of ZnS and CdS samples to those of the ZnCd QDs. The Figure also shows important biological reactions corresponding to the standard redox potentials and band gap energies of the semiconductors. This comparison demonstrates that the photogenerated ZnCd QDs can be used for the same reactions as those of classical bulk ZnS or CdS. It was previously shown that QDs form a conjugate with biomolecules, such as proteins [47,48] and DNA [49], and generate complex structures. UV tuning of these complex structures could lead to systems resembling photochemical reaction centers as schematically shown in Figure 2b (right).

Other features of nanoparticles include the ability to concentrate important substances on their surface by covalent or electrostatic interactions, and surface radical reactions can be controlled based on of UV radiation. A frequently stated advantage of radical-based methodologies for molecular formations is that radical reactions are essentially free from solvent effects on their reaction kinetics and, hence, on the reaction products [50]. Radicals cause also polymerizations or bond cleavage [51,52]. Catalytic conversion of solar to chemical energy has also been proven on plasmonic metal nanostructures [53]. The main advantages (preconcentration of substances on particle surface by a covalent bond or electrostatic interaction, redox reaction controlled by redox potential or light, UV protection by absorption of radiation, energy conversion by Förster resonance energy transfer or plasmonic resonance and complex structures formations) of nanoparticles in the origin of life context are shown in Figure S4b.

As mentioned above, nanoparticles can participate as catalysts in a several of specific (nanozymatic) or non-specific reactions that are relevant to prebiotic chemistry and therefore could be co-assisted with the formation of primitive prebiotic photodynamic redox chemical networks with proto-enzymatic activity and could be progressed into the metabolic-like networks and life (protocell) on early Earth.

**Figure 3:**
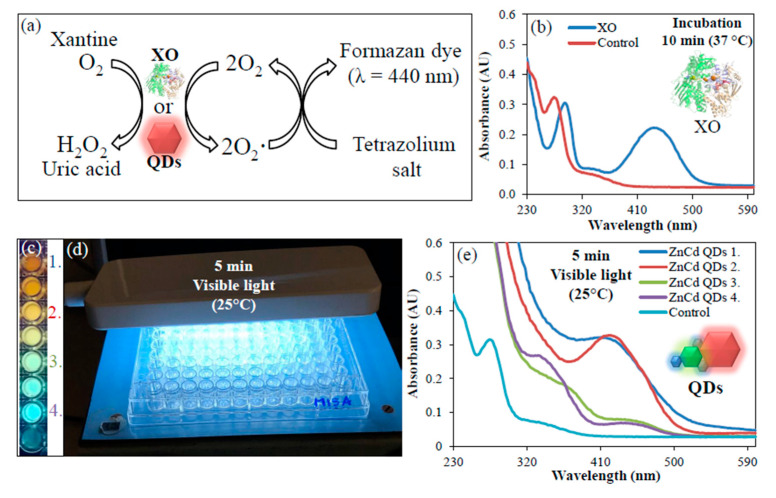
XO-like activity of ZnCd QDs. (**a**) Scheme of the XO activity (**b**) Absorption spectra of the XO substrate (blue trace) and distilled water (red trace) incubated with XO for 10 min at 37 °C (**c**) ZnCd QDs selected for monitoring of XO-like activity from Figure 2a (**d**) Photograph of visible light irradiation of mixture of ZnCd QDs with XO substrate (**e**) Absorption spectra obtained from XO substrate mixed with ZnCd QDs and irradiated for 5 min by visible light.

Above, we have shown that the UV-assisted (one-pot) reaction leads to the formation of ZnCd QDs (blue to red emission) from a simple mixture of precursors. This type of nanomaterial (ZnCd QDs), however, mimics the function of the enzyme xanthine oxidase (XO). XO commonly catalyzes the oxidation of hypoxanthine to xanthine and can further catalyze the oxidation of xanthine to uric acid. XO plays also an important role in the catabolism of purines. In our experiments, we monitored the rate of the reduction with O_2_ related to the XO activity as well as to ZnCd QDs activity (mimic XO) (Figure 3a). In Figure 3b, the absorption spectra of the substrate of XO (λ = 440 nm) after 10 min incubation with XO at 37 °C is shown. Next, from the previous experiment, we chose four samples of QDs labelled as ZnCd 1., ZnCd 2., ZnCd 3. and ZnCd 4. (Figure 3c). These QDs were mixed with the substrate and an irradiated by visible light for 5 min (Figure 3d). The XO-like activity was manifested by increase in absorption signal at λ = 440 nm (Figure 3e). Individual precursors of ZnCd QDs were also tested (Figure S5). Only ZnCd QDs (prepared by 2 min UV irradiation) exhibited XO activity.

The properties of nanoparticles position them to a role of a plausible candidate chemical system which might have participated on a transition from abiotic chemistry to a more complex proto-metabolic world. However, it is not known how this transition happened. In fact, even the most accepted theories cannot answer this fundamental question. What happened on Earth in the critical period between the emergence of liquid water ca. 4.32 Gya, probably coinciding with the last ocean vaporizing impact [54,55,56] and origin of the first living system on Earth prior to 3.8 Gya [57], 3.95 Gya [58], 4.1 Gya [59] and speculatively as early as 4.28 Gya [60]. Due to the lack of a well-preserved geological rock record, the physical and chemical environment at this time is obscured and the prevailing conditions available to Hadean prebiotic synthesis and origin of life are uncertain. Although it is very hard to complete diverse studies to one unified picture, we can point several arguments demonstrating the plausibility and limitations of chemistry described above in this paper. It is known that early Earth crust was enriched by highly siderophile elements during the later veneer. The concentration Cd and Zn in chondritic impactors can range between 0.1 to 686 ppb and from 0.04 to 2100 ppm respectively [61,62]. Cd is a relatively volatile element and it is strongly depleted in all iron meteorite groups and relatively abundant in primitive carbonaceous and E4 chondrites [63]. In opposite, Zn is a typical trace element both in iron meteorites and silicate material of chondrites. We can assume that Cd and Zn, together with other heavy elements, can be released by water alteration of impact structures. Evidences of water alteration and hydrothermal environment have been discovered, for instance, in the case of the famous Gale Crater formed on Mars during the late heavy bombardment about 3.8 Gya, close to the time of origin of life on Earth [64]. Importantly, Zn enrichment 4000–8000 ppm in sandstone discovered in sedimentary rocks in Gale Crater, Mars, by the Alpha Particle X-ray Spectrometer on the rover, Curiosity, indicates such a fractionation connected to hydrothermal fluids [65]. However, information regarding the concentration of Cd in the Gale impact structure is missing in current literature. Enrichment in Zn and probably also in Cd (which is, however, truly less abundant) connected to impact structures and subsequent hydrothermal activity very likely exists. However, its maximum degree as well as composition of fluids and also possible interference with other elements must be explored in future. Regarding an important agent for nanoparticle laboratory synthesis, mercaptosuccinic acid (MSA), it is not known how specifically this molecule can at least appear in the early planetary environment and reach plausible concentrations. It is also not known if this compound can be substituted by another plausible molecule. However, several sulfur-containing molecules have been detected in the Gale impact structure on Mars [66] and the introducing of very complex sulfurcontaining organic compounds in prebiotic chemistry has been extensively explored starting with Wächtershäuser’s hypothesis of the iron-sulfur world [67]. Acetic acid, whose Cd and Zn salt was used for the reaction described in this study, is also a common compound in Fischer-Tropsch processes and Wächtershäuser’s prebiotic chemistry [68,69]. The neutral pH used for laboratory preparation of nanoparticles in this study slightly differs from typical pH of hydrothermal fluids, which is, however, slightly acidic on current Earth (5.1–5.4) [70]. However, pH of hydrothermal fluids depends on the chemical composition of the environment. For instance, nanometer-sized silica particles detected by the Cassini Cosmic Dust Analyzer are found to have originated from the hydrothermal environment on Saturn’s moon, Enceladus. The presence of these particles provides tight constraints on the particular conditions of the interior ocean: high-temperature reactions (≥∼90 °C), moderate salinity (≤∼4%), and notably, alkaline seawater (pH = 8.5–10.5) [71]. Really, it has been found that in several terrestrial silica-undersaturated, alkaline host rocks, pH increases to 7 and above. The pH in the early environment might have been also stabilized by phosphate buffer similarly with our experimental arrangement. The Recent study of Ritson, Mojzsis and Sutherland demonstrates the formation of PO_4_^3−^ anion from phosphorus species upon ultraviolet light in the presence of H_2_S/HS^−^ [72]. These experiments show that PO_4_^3−^ was widely available to prebiotic chemistry and nascent life on early Earth and potentially on other planets. It should be noted that our previous work demonstrates that water iron-rich smectites likely produced by water alteration of meteoritic material are plausible catalysts for the thermal synthesis of canonical nucleobases, hypoxanthine, purine, urea and glycine [29]. The synthetic potential of formamide as a prebiotic feedstock molecule was reported by the Saladino and Di Mauro groups and confirmed by other studies [73].

Based on these results, it can be tentatively postulated that post-impact hydrothermal environment on early planets rich in heavy elements involving also Zn and Cd, sulfur compounds, phosphates and organic compounds, was plausible for the prebiotic synthesis of feedstock molecules, as well as for the origin of protometabolic pathways including the mechanism described in this study. The next step of chemical evolution could have been associated with nanozymatic activity of different nanoparticles that likely formed primitive prebiotic photodynamic redox chemical networks with proto-enzymatic activity. It is a challenging topic for future studies to combine diverse results into one picture showing if and how such a scenario of the origin of life happened. It is also very likely that important evidences will be provided by future explorations of Gale Crater on Mars.

## 4. Conclusions

In this article, the experimental and theoretical evidence confirming UV-induced formation of ZnCd QDs was presented and its potential role in prebiotic synthesis was suggested. The main abilities of ZnCd QDs include: (a) ZnCd QNPs are formed by redox reactions during photooxidation from thiol containing compounds and metal ions (e.g., Zn(II), Cd(II)) in a short time), (b) ZnCd QDs exhibit strong fluorescence (QY = 47%), (c) ZnCd QDs grow during UV irradiation. Based on this, it was proposed to call these redox reactions (ZnCd QDs formation and tune-up) a photodynamic redox system.

Moreover, the formation of these nanoparticles on the early Earth is assumed. All the chemical substances necessary for such kind of synthesis have been very likely in Hadean environment. All ZnCd QDs precursors have been delivered by extraterrestrial impacts, volcanic activities or hydrothermal vents and released by subsequent alteration of impact structures or survived impactors. Upon strong UV radiation (verified by stratospheric experiment), metal nanoparticles could have formed primitive prebiotic photodynamic redox chemical networks with proto-enzymatic activity. Tuning of these photodynamic redox systems and conjugation with molecules or other nanostructures has fascinating biologically relevant activity and could progress into the metabolic-like networks and life on early Earth.

## Supplementary Materials

The following are available online at https://www.mdpi.com/2079-4991/10/8/1529/s1, Figure S1: Precursors (Zn:Cd:MSA in phosphate pH 7) were irradiated by UV for 5 min and fluorescence mapping was performed in excitation range 240–380 nm and emission spectra of ZnCd QNPs were recorded in range 400–680 nm, Figure S2: Band gap tuning (emission properties at λex = 250 nm in emission range 300–800 nm, manifested as a red shift) of ZnCd QDs containing (a) Zn(II), Cd(II) and 4 mM MSA in phosphate buffer pH 7 after 2 (blue), 4 (red), 8 (green), 16 (purple) and 32 (light blue)-minute UV irradiation and/or (b) ZnCd QDs containing Zn(II), Cd(II) and 2 (light blue), 4 (purple), 8 (green), 16 (red) and 32 (blue) mM MSA in phosphate buffer pH 7 after 4 min UV irradiation. (c) Schematic illustration of the change in energy levels as a function of QDs size, Figure S3: (a) Schematic representation of balloon stratospheric platform with six cuvettes attached under the probe (exposed UV) and two cuvettes placed in the probe (unexposed UV). (b) The trajectory of flight with duration approximately of 150 min. (c) ZnCd QDs emission spectra at λex = 250 nm in range 300–800 nm, recorded after landing of stratospheric (balloon) platform, Figure S4: (a) The comparison of standard redox potential (V) with band gap energy (eV) of standard inorganic ZnS, CdS and proposed ZnCd QDs and important biological reactions corresponding to standard redox potential and band gap energy. (b) Schematic representation of solar driven (formation) nanoparticles and their features in origin of life context. Nanoparticles are able to participate as catalysts in a number of specific (nanozymatic) or non-specific reactions that are relevant to prebiotic chemistry and therefore could be co-assisted with the formation primitive prebiotic photodynamic redox chemical networks with proto-enzymatic activity and could progressed into the metabolic-like networks and life (protocell) on early Earth, Figure S5: Absorption spectra obtained from XO substrate mixed with ZnCd QDs precursors and irradiated for 0 min (a) and for 5 min (b) by visible light. Table S1: First 96 Well Plate, Table S2: Second 96 Well Plate, Video S1: Formation of fluorescent nanoparticles by UV irradiation.
